# After the spotlight: are evidence-based recommendations for refeeding post-contest energy restriction available for physique athletes? A scoping review

**DOI:** 10.1080/15502783.2022.2108333

**Published:** 2022-08-08

**Authors:** Sara Chica-Latorre, Claire Buechel, Kate Pumpa, Naroa Etxebarria, Michelle Minehan

**Affiliations:** University of Canberra, Research Institute for Sport and Exercise, Canberra, Australia

**Keywords:** Physique contest, bodybuilding, recovery, metabolic adaptation, dietary recommendations, body composition

## Abstract

Background: To date, there is limited consensus on post-contest recovery recommendations for natural physique athletes. The available literature emphasizes the negative consequences of extreme dieting associated with physique contests, yet offers only speculative suggestions to facilitate physiological recovery post-contest. This scoping review evaluates evidence-based recommendations for recovery in post-physique contests. Methods: The online search engines and databases Google Scholar, PubMed, and Scopus were searched systematically and 12 peer reviewed journal articles were included in the review. Results: Six key factors were identified that directly impacted on physiological recovery post-contest: (1) body composition, (2) recovery dietary intake, (3) resting metabolic rate (RMR) restoration, (4) endocrine measures recovery, (5) menstrual cycle recovery, and (6) psychological aspects of recovery. Conclusions: Three dietary strategies have been proposed to facilitate physiological recovery post-contest while bearing in mind body composition and future athlete outcomes; (1) a gradual increase in energy intake to maintenance requirements, (2) ad libitum eating, (3) an immediate return to maintenance energy requirements. Future research is required to determine the timeline in which full physiological recovery occurs post-contest and which strategies best support athlete health and performance during post-contest recovery.

## 1. Background

”Physique contest” is an umbrella term used that refers to bodybuilding/modeling events and typically encompasses a broad spectrum of categories for amateur and professional athletes, such as bikini modeling, fitness modeling, physique and figure contests, and traditional bodybuilding contests [1,2]. Physique contests could also further bifurcate into natural (drug-free) or non-natural (with performance and appearance enhancing drug use) physique contests [3]. For natural physique contests worldwide, several anti-doping governing bodies have arisen, such as the International Federation of Bodybuilding (IFBB) and the amateur athlete equivalent; the National Physique Committee (NPC). Each governing body offers different competition categories and enforces different rules for competition [3]. The present scoping review addresses natural drug-free physique contests.

Physique contests are based on an athlete’s aesthetics and judged according to the athlete’s muscularity, shape, and symmetry (subject to competition category), and stage presence and presentation, as stated by the NPC [1]. These contests are characterized by a rigorous preparation phase aiming to achieve low-fat mass, with body fat percentages generally ranging between 4% and 6% for males and 10% and 13% in females, while retaining lean mass [4–6]. Physique athletes typically periodize their training year into four distinct phases: contest preparation, a short peaking phase pre-contest, post-contest recovery, and the off-season or hypertrophy-phase [7]. Preparation and peaking phases involve energy restriction (energy deficit of 20% or more), coupled with nutrient manipulation and increased exercise output, to minimize body fat and optimize muscle mass [7–10]. The recovery phase involves restoration of fat mass and reversing the negative physiological and psychological adaptations that may have occurred during the preparation phase, such as reduced resting metabolic rate (RMR), lean mass loss, hormonal imbalances, reduced performance, and hedonic responses to food [7]. The off-season focuses on gaining muscle mass to improve physique outcomes for future contests [11]. Due to the scarce evidence-based research available, nutrition guidelines for the different phases of physique training have, until recently, relied largely on anecdotal evidence from applied experience (i.e. bodybuilding and strength and conditioning peers’ and coaches’ experiences, and related internet sites) [12]. In recent years, as the popularity of physique contests has increased, new evidence-based research has worked to close the gap between practice and evidence, and to provide supportive nutrition guidelines for physique athletes [4,7,9].

During the contest preparation phase, current recommendations include gradual energy restriction, protein increments, and carbohydrate manipulation to achieve the characteristic lean body composition of physique athletes [9,10,13,14]. Specifically, these recommendations detail implementing an energy restriction resulting in 0.5–1% total body mass loss per week, prioritizing protein intake of 2.3 to 3.1 g.kg^−1^ of lean body mass, maintaining fat intake between 15% and 30% of the total calories, and filling in remaining total calories with carbohydrate [9]. Protein and carbohydrate intake are prioritized around training sessions to minimize muscle loss during energy restriction and support energy levels during training [9]. A key strategy to sustain lean mass and maximize body fat loss during contest preparation is to consume a high protein diet to mitigate muscle catabolism induced by weight loss [8,9]. The risk of muscle catabolism increases if exposed to rapid weight loss, regardless of protein intake and adherence to resistance training regimes [15]. Hence, longer preparation phase regimes of 12 to 30 weeks and controlling weight loss to less than 1% of body mass per week, may reduce risk of muscle loss and positively benefit an athlete’s final contest physique and their ability to recover post-contest to restore baseline physiology [8,9]. Moreover, increases in muscle mass have been observed in some physique athletes who undergo longer preparation phases while maintaining a high-protein intake, even while in a significant energy deficit [16]. This highlights the benefits of physique athletes choosing a preparation phase length that is suitable for their starting body composition to allow for 1% body mass loss per week and reach a low body fat percentage for contest day with minimal to no muscle loss.

Regardless of the length of the contest preparation phase, as energy availability decreases and body fat reduces, metabolic adaptations occur to restore baseline body mass [17]. These metabolic adaptations involve hormonal changes, particularly testosterone, estrogen, thyroid hormones (T3 and T4), ghrelin, insulin, and cortisol, along with a reduction of RMR [10,17–19]. These adaptations result in weight loss “plateaus,” requiring further energy restrictions through dietary intake or exercise expenditure to continue the energy deficit for fat loss [17]. However, the consequences of extreme energy deficits can lead to low energy availability (LEA), where the body does not have enough energy to support optimal physiological function and metabolic systems are disrupted [20]. Muscle loss, hormonal imbalances, decreased immune function, and negative psychological outcomes have been shown in bodybuilders suffering from LEA [21]. Outside physique contests, similar metabolic disruptions have been found in untrained and overweight or obese populations seeking to reduce body fat [22–24]. It is suggested that these adaptations favor weight regain due to decreases in energy expenditure, fat oxidation, and hormonal shifts to promote return to baseline weight [22].

The post-contest recovery phase consists of athletes increasing energy availability to restore baseline physiology and repair psychological aspects of restrictive dieting experienced during the preparation phase [7]. Immediately post-contest, hedonic responses to food are common, creating a high potential for fat or weight overshooting, where an athlete rapidly increases body mass post-contest above their original baseline body composition prior to contest preparation [25]. This weight overshooting appears to be associated with the restoration of hormonal balance and metabolic function of the athlete [7,12,26,27]. However, rapid changes in body composition post-contest can affect competitors not only physiologically but also psychologically due to unwanted increases in fat mass [7,17,26,28]. Hence, adequate dietary interventions and recommendations to guide post-contest recovery are imperative to support athlete physical and psychological health. There appears to be no clear consensus in the existing literature regarding post-contest recovery methods, other than ongoing evidence that it is common for physique athletes to rapidly increase energy intake post-contest ad libitum [7]. Anecdotal evidence suggests that a controlled gradual restoration of energy intake toward adequate energy availability is a possible tool to minimize fat mass gain [7,11], bringing about the concept of ”reverse dieting.” Given the absence of comprehensive recommendations around post-contest recovery practices, it is common for physique athletes to rely on anecdotal evidence to achieve energy restoration in the post-contest recovery phase [10,11]. The length of the recommended post-contest recovery phase is currently unclear, with some athletes preferring a rapid ad libitum energy intake and some applying a more structured gradual approach to increase energy intake [7]. Therefore, the aim of this scoping review is to summarize the strategies of post-physique contest recovery and characterize the timeline in which physiological restoration occurs.

## 2. Methods

### 2.1 Inclusion criteria

This scoping review was conducted according to the PRISMA-ScR protocol [29], and the PICO search framework [30].

Electronic web search engines and databases including Google Scholar, PubMed, and Scopus were searched from January 2000 to August 2021. The search strategy consisted of the following terms: Physique athlete AND Metabolic adaptation AND Dietary intervention AND Refeeding AND Contest preparation AND Contest recovery as well as associated MESH terms. Sequentially, titles and abstracts were scanned, with relevant articles proceeding for a full-text review. Additionally, text words and reference lists from relevant papers were screened for further search of relevant articles. Two authors (SCL, CB) screened articles with a third author (MM) acting to resolve disputes if required.

The search was restricted to human studies published in English. The search included the following criteria: (1) natural adult female and/or male physique contest athletes, 18 years and over; (2) acute or long-term interventions; (3) peer-reviewed articles, such as quantitative and/or qualitative studies, randomized control trials, and case studies; and (4) literature including findings and discussion relevant to the post-contest recovery phase. The past two decades have shown an increased popularity of drug-free natural physique contests, a progressive globalization of the bodybuilding industry, and the introduction of new competition categories welcoming competitive and recreational participants, which reflects an expansion in the literature focusing on physique athletes. Furthermore, the World anti-doping agency (WADA) formed in November 1999, which allowed the formalization of sanctioned drug-testing in the sport [3].

**Figure 1.**
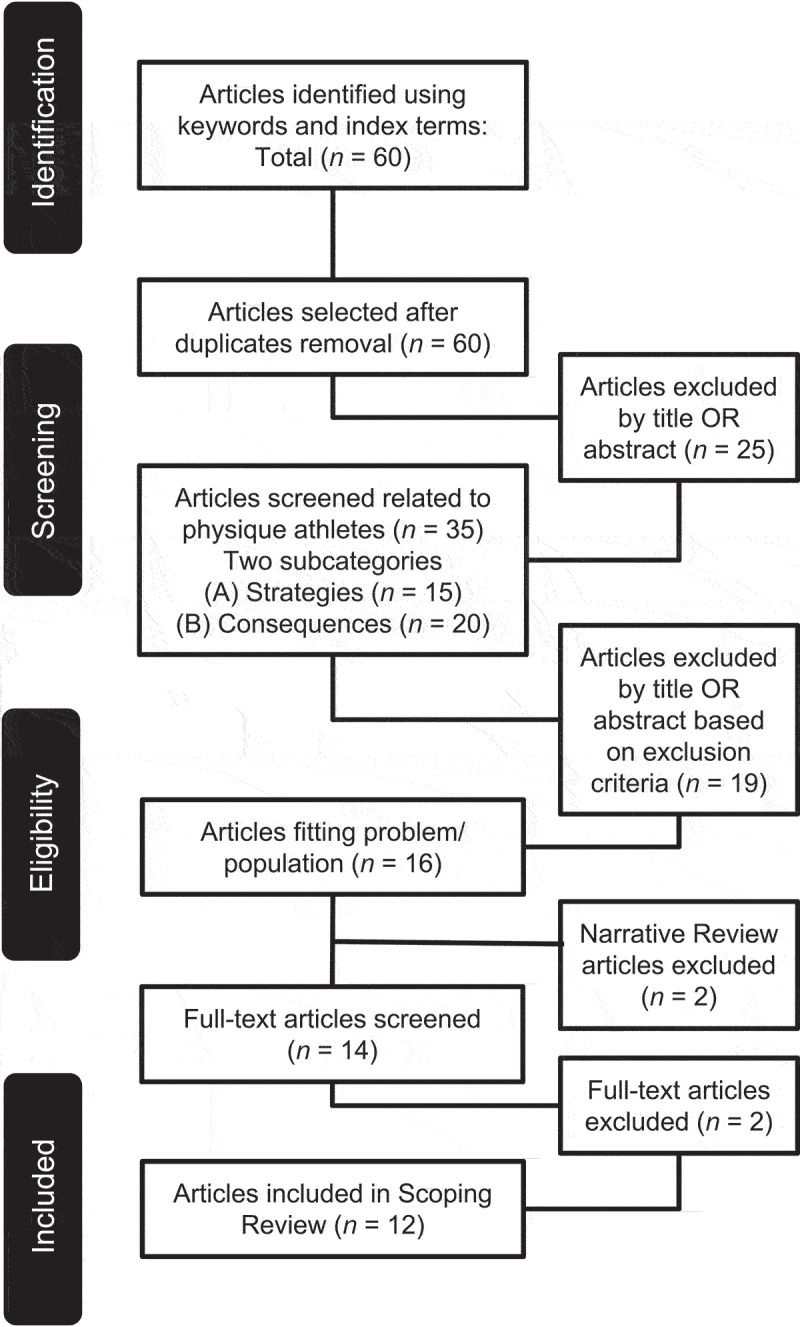
PRISMA-ScR flow chart for article identification, inclusion, exclusion, and selection.

Articles were excluded if: physique athletes were not involved in the data; dietary measures, assessments, recommendations, or nutrition-associated consequences related to post-contest recovery were not included; or participants of studies were identified to be using androgenic anabolic steroids to avoid any confounding links toward misleading advantageous dietary strategies [31]. The article selection process is summarized comprehensively in Figure 1.

### 2.2 Data charting process

An Excel data charting tool was created to track the selection process. Data charting was developed by the two main authors (SCL, CB) of this scoping review. Authors SCL and CB individually cross-checked selected articles to guarantee accurate inclusion of relevant articles and secure consistency in evidence-based rationale toward evaluations and future research recommendations.

### 2.3 Article selection

A total of 60 articles were identified from the initial search and their title and abstract screened for inclusion. There were no duplicate articles during the search process. For this initial screening, a total of 35 articles were related to physique athletes. Out of the 35 articles, 19 articles were excluded as they lacked information within the abstract relating to post-contest recovery refeeding. Sixteen articles fit the inclusion criteria. Out of the 16 articles, two were narrative reviews, hence excluded from the search. The full text of 14 articles were then independently reviewed by two authors (SCL, CB) with additional two articles excluded due to their findings being irrelevant to the question of this review. Twelve articles were selected and included in this scoping review (Figure 1). The characteristics of the selected studies are summarized in Table 1.

**Table 1.** Summary of data charting for selected studies on post-contest recovery.

Reference	Study design	Scope	Aspects of ‘post-contest recovery’ addressed	Main findings	Comments
Chappell, Simper, Trexler, & Helms 2021	Prospective case-series	Biopsychosocial changes in 3 male and 1 female amateur physique athletes (30-44 yrs) over 8 months; 6 months pre-contest and 2 months post-contest.	Diet and exercise strategies observed, estimated energy availability, RMR (indirect calorimetry), cardiovascular measures, body composition (BIA, ultrasound), blood and saliva markers, exercise performance, and psychological assessments.	**Metabolic rate**: ↑ RMR toward baseline post-contest, *n* = 2 exceeded baseline RMR.**Body composition**: ↑ FM.**Hormones**: T resolved coinciding with ↑ FM, EA and anaerobic performance.**Exercise performance**: ↑ EP post-contest.	Authors suggest to better retain FFM, ↑ EA (> 30 kcal/kg BM) by either manipulating training load or ↑ energy intake.Rapid ↑ BM and ↑ FM post-contest likely assisted recovery speed of physiological variables.
Halliday, Loenneke, & Davy 2016	Retrospective case study	Changes in dietary intake, body composition, and menstrual cycle of an amateur female figure competitor (26 yrs) across 40 weeks; 20 weeks pre- and post-contest. Additional measurements collected 1 year post-initiation of study and when menses returned 71 weeks post-contest.	Diet and exercise strategy observed, energy availability estimated, body composition (weight, DXA, skinfolds), and menstrual cycle.	**Diet**: ↓ Energy intake pre-contest and gradual ↑ energy intake post-contest (↑ CHO and fat, protein remained above 2.0 g/kg)**Body composition**: ↑ BM in proportion to energy intake post-contest. Skinfolds returned to baseline 20 weeks post-contest. Body composition and BM at 32 and 71 weeks post-contest > composition previously maintained for years leading up to competition.**Energy availability**: ↑ EA 10 weeks post-contest.**Menstrual cycle**: Resumption of menstrual cycle at 71 weeks post-contest.	Participant began study at 15% BF.Authors suggest EA declines and body composition modifications pre-contest could have persistent, damaging consequences on reproductive hormonal profiles.Gradual ↑ energy intake post-contest while maintaining high protein intake may minimize fat accumulation following energy restriction.
Hulmi et al. 2017	Prospective cohort study	Physiological effects of 27 female amateur physique athletes (27.2 ± 4.1 yrs) against 23 control participants (*n* = 50) across 9 months; 5 months pre-contest and 4 months post-contest.	Diet and exercise strategies observed, body composition (BIA, DXA, skinfolds, ultrasound), exercise performance, cardiovascular measures, serum hormone markers, psychological questionnaires.	**Diet**: ↓ Energy intake pre-contest and ↑ energy intake toward baseline in recovery period.**Body composition**: ↔ LBM. BMI and bone mass returned to baseline post-contest.**Hormones**: ↓ Leptin, T, and E2 pre-contest. ↑ Leptin and E2 to baseline 4 months post-contest. T and T3 remained below baseline.**Menstrual cycle**: Physique competitor group ↑ menstrual irregularities than control group (amenorrhea: *n* = 3 at baseline, *n* = 7 at final data point).	Most competitors (*n* = 19) reported using E2-containing contraception.Authors suggest visceral fat mass may be related to menstrual function. ↓ FM in these areas combined with energy deficit may signal hypothalamus-pituitary axis to ↓ hormones relating to ovulation such as E2 and lead to hindering ovulation and menstrual bleeding.Longer recovery period required for full recovery of T3 and T.
Lapinskienė, Mikulevičienė, Laubner, & Badaras 2018	Case report	Outcomes of a male (28 yrs) professional bodybuilder admitted to emergency hospital 2 days after physique contest.	Diet strategy noted, cardiovascular measures, blood panel, and grip strength. Occurrence of refeeding syndrome.	**Diet**: Gradual ↓ CHO 4 months pre-contest, eliminated all CHO 4 weeks pre-contest. Fast-acting CHO consumed on competition day and post-contest.**Body composition**: ↓ BM pre-contest.**Blood panel**: Refeeding syndrome; hypokalaemia, hypophosphatemia, ↓ Mg, hyperglycemia, ↑ CK, ↑ LFTs.**Recovery**: 4 days in hospital care before discharged.	BF% above the low EA threshold (4-5% BF in males and energy intake >30 kcal/kg of FFM), may be protective against refeeding syndrome in this population.
Longstrom et al. 2020	Prospective case-series	Physiological, psychological, and performance-related changes in 3 male (34 ± 6.8 yrs) and 4 female (29 ± 4.9 yrs) natural physique athletes; observed from 2 weeks pre-contest to 10 weeks post-contest.	Diet strategies observed, Body composition (BIA, skinfolds), psychological aspects, sleep quality, menstrual irregularities, RMR (indirect calorimetry), exercise performance, and serum hormone markers.	**Diet**: *n* = 3 adhered to reverse dieting approach (gradual ↑ energy intake while maintaining cardiovascular training), with minimal BM gain and RMR changes, and small ↓ FFM; inadequate to improvements physiological outcomes.**Hormones**: ↑ T3, T4 and leptin 10 weeks post-contest.**Body composition**: ↑ BM and FM. 1 female ↑ calorie intake by 97% coinciding with largest ↑ BM (22%). Other participants ↑ BM by <10%.**Metabolic rate**: ↑ RMR in most participants except for 1 female with ↓ RMR (suggested due to exercise induced amenorrhea) and 1 male (↔ despite ↑ energy intake).**Menstrual cycle**: All females experienced irregularities post-contest. 1 female normalized menstrual function post-contest (greatest ↑ energy intake, BM, FM).**Exercise performance**: ↑ EP post-contest.	**Limitations**: No baseline measurements prior to contest preparation.Authors suggest recovery phase should consist of enough energy intake for BF gain, while avoiding BF overshooting. Longer off-season should be considered to allow for physiological recovery and support improvements to LBM and metabolic rate for future physique contest success.Some physiological measures may improve within weeks; others may remain downregulated for upwards of 9 months.↑ FM associated with ↑ RMR and leptin.
Mitchell, Slater, Hackett, Johnson, & O’connor 2018	Prospective case-series	Physiological changes in 9 male physique athletes (29 ± 9.5 yrs) over 20 weeks; 16 weeks pre-contest and 4 weeks post-contest.	Diet and exercise strategies observed, Body composition (BIA, DXA, skinfolds), cardiovascular measures, hormone markers, and RMR (indirect calorimetry).	**Diet**: ↑ Energy intake above baseline post-contest (↑ CHO and fat).**Hormones**: ↑ T and IGF-1 toward baseline. ↑ C and leptin. Hormones resolved 4 weeks post-contest.**Body composition**: ↑ FM toward baseline.**Metabolic rate**: ↔ RMR.	**Limitation**: RMR may have been inflated due to limited exercise-free period pre-testing (metabolic rate may remain elevated for up to 48 hrs following resistance exercise).Authors suggest maintenance of LM pre-contest may have contributed to ↔ RMR.
Newmire & Webb 2021	Prospective case-series	Physiological and psychological changes in 2 female physique athletes (32 and 44 yrs) across 20 weeks; 16 weeks pre-contest and 4 weeks post-contest.	Body composition (DXA, BIA, ultrasound), RMR (indirect calorimetry), and psychological assessments.	**Diet**: 32 yrs athlete followed reverse dieting protocol and gradually ↓ exercise frequency and volume.**Body composition**: ↑ BF%.**Metabolic rate**: ↑ RMR.	Diet, exercise, and hormone markers were not followed in the recovery phase.The 32y/o athlete moved into recovery phase post-contest and the 44y/o athlete continued preparation for a subsequent competition.
Pardue, Trexler, & Sprod 2017	Prospective case study	Physiological effects of an amateur male bodybuilder (21 yrs); observed for 13 months; 8 months pre-contest and 5 months post-contest	Diet and exercise strategy observed, serum hormone markers, body composition (bod pod, DXA), exercise performance, RMR (indirect calorimetry), and sleep quality.	**Diet**: Reverse dieting strategy; gradual ↑ calorie intake (10-30 g CHO and/or 4-10 g fat added per week), ↓ exercise.**Hormones**: All measured hormones except for leptin, reverted toward reference range post-contest. Leptin remained low throughout study.**Body composition**: ↓ BF% pre-contest, ↑ BF% post-contest to baseline.**Exercise performance**: power output was not fully restored post-contest.**Metabolic rate**: ↑ RMR, not fully restored to baseline.	Authors suggest reverse dieting strategy was used to facilitate recovery while minimizing binge-eating and rapid fat gain that are anecdotally observed post-contest in physique athletes.
Rossow, Fukuda, Fahs, Loenneke, & Stout 2013	Prospective case study	Physiological profile of a male (26 yrs) professional bodybuilder over 12 months; 6 months pre- and post-contest.	Cardiovascular measures, body composition (BIA, DXA, Bod Pod), RMR (indirect calorimetry), exercise performance, serum hormone markers, and psychological aspects.	**Diet**: ↑ Energy intake post-contest (↑ fat and CHO).**Cardiovascular**: HR and BP returned to baseline post-contest.**Body composition**: BF% returned to baseline within 6 months post-contest.**Exercise performance**: Strength did not fully recover.**Hormones**: ↓ T pre-contest and ↑ baseline post-contest.**Psychological**: ↑ Mood disturbance pre-contest and recovered 6 months post-contest.	**Limitations**: Serum hormones were measured every 3 months; hormones may have recovered sooner than interval demonstrated.
Schoenfeld et al. 2020	Prospective case study	Physiological profile a male (25 yrs) amateur bodybuilder over 12 months; 8 months pre-contest and 4 months post-contest.	Diet and exercise strategy observed, body composition (BIA, skinfolds, ultrasound), RMR (indirect calorimetry), cardiovascular measures, exercise performance, serum hormone markers, and eating behaviors.	**Diet**: ↑ energy intake immediately post-contest and ↓ energy intake by final data point.**Body composition**: ↑ BM post-contest. BF <10% for length of study. Muscle thickness and LBM returned to baseline 2 months post-contest.**Metabolic rate**: ↑ RMR post-contest, exceeded baseline 2 months post-contest.**Psychology**: Fluctuating cognitive restraint and uncontrolled eating scores, returned to baseline 4 months post-contest.**Hormones**: ↓ T, SHBG, T3, AST, ALT, BUN, WBC, AGR pre-contest, returned to baseline within 30 days post-contest.	Authors note a refeed strategy (temporary ↑ CHO) was used throughout contest preparation and did not attenuate unfavorable metabolic and physiologic effects of BM loss.**Limitations**: Participant started study already lean (9% BF).
Tinsley et al. 2019	Prospective case study	Body composition and neuromuscular performance in a female natural figure competitor (27 yrs) across 8 months (34 weeks); 18 weeks pre-Contest 1, a 7 week interim until Contest 2, and a 9 week recovery period.	Diet and exercise strategies observed, body composition (BIA, DXA), cardiovascular measures, RMR (indirect calorimetry), exercise performance, and eating behavior questionnaire.	**Diet**: Gradual ↓ energy pre-contest. Post-contest, energy intake restored in a ‘stepwise’ manner until reaching baseline BM (reverse dieting). A high-protein diet was followed for duration of study.**Body composition**: ↓ FM, ↑ FFM pre-contest. Post-contest, ↑ BM by 1% per week (reached baseline at 9 weeks post), ↑ FM by 8.1-9.2% per week, ↑ FFM exceeded baseline.**Metabolic rate**: ↑ RMR to baseline 1 month post-contest, exceeded in week 9.**Eating behavior**: ↑ Uncontrollable eating scores post-contest, trended down toward baseline by week 9.**Menstrual cycle**: ↔ Nil regular menstrual cycles (1 menstrual period 3 months before first competition).	Participant began study at 20.3% BF.Authors suggest high protein intake during recovery phase might have facilitated RMR recovery.
Trexler, Hirsch, Campbell, & Smith-Ryan 2017	Prospective case-series	Physiological changes in 8 female and 7 male natural physique athletes (18-50 yrs); 1 week pre-contest to 6 weeks post-contest.	Diet strategies observed (diet logs), Body composition (BIS, ultrasound, skinfolds), RMR (indirect calorimetry), and hormonal profile via saliva.	**Diet**: ↑ energy intake post-contest (↑ CHO and fat).**Body composition**: ↑ BF%, ↑ BM.**Metabolic rate**: ↑ RMR positively associated to ↑ BF% and protein intake.**Hormones**: ↑ T and C. ↔ Ghrelin and leptin.	Authors note RMR suppression is highly variable between individuals.Post-contest preferential fat gain may be minimized and lean mass restoration maximized by implementing robust resistance training, a modest caloric surplus, and relatively high protein intake following competition to facilitate recovery.

Variations denoted as ↑ (increase); ↓ (decrease); or ↔ (no change). CHO = carbohydrate; LBM = lean body mass; BM = body mass; FFM = fat free mass; BF = body fat; HR = heart rate; BP = blood pressure; T = testosterone; E2 = estradiol; C = cortisol; SHBG = sex hormone binding globulin; T3 = triiodothyronine; T4 = thyroxine; Mg = magnesium; AST = aspartate transaminase; ALT = alanine aminotransferase; BUN = blood urea nitrogen; WBC = white blood cell count; AGR = albumin globulin ratio; IGF-1 = insulin-like growth factor-1; LFT = liver function tests; CK = creatine kinase; FM = fat mass; EA = energy availability; EP = exercise performance; yrs = Years.

## 3. Results

### 3.1 Profile of studies reviewed

Of the 12 included articles, all were case studies observing one or more athletes across physique contest phases. Most articles reviewed were published between 2018 and 2021 (58%, *n* = 7). Six studies (50%) reported on one individual athlete, five studies (42%) followed 2 to 15 athletes, and one study observed a total of 50 participants, 27 of which were physique athletes, and the remaining were a non-competing control group. Across all the reviewed studies, the total number of physique athletes observed post-contest was 70 (*n* = 26 male, *n* = 44 female). Case study and case series populations ranged in age from 20 to 50 years. The articles were divided relatively evenly between mixed male and female studies (33%, *n* = 4), exclusively female (25%, *n* = 3), and exclusively male (42%, *n* = 5). Most studies following physique competitors post-contest were limited in duration, with four studies (33%) collecting data for less than 2 months, and six studies (50%) collating data between 2 and 6 months post competition. There were six major factors identified that had a direct impact on physiological recovery post-contest: (1) body composition, (2) dietary intake, (3) resting metabolic rate (RMR), (4) endocrine measures recovery, (5) menstrual cycle recovery, and (6) psychological aspects of recovery.

### 3.2 Body composition

Body composition was reported in 92% (*n* = 11) of the case studies, with measures including bioimpedance analysis (BIA), bioimpedance spectroscopy (BIS), amplitude-mode ultrasonography, dual-energy x-ray absorptiometry (DXA), air-displacement plethysmography, and skinfold assessments. All case studies measuring body composition employed at least two of these techniques, with a maximum of four. The total number of participants measured for body composition in the post-contest period was 69 (*n* = 25 male, *n* = 44 female). For case studies that observed athletes for up to 2 months post-contest (*n* = 4), an increase in fat mass toward pre-contest preparation baseline body composition was observed in all athletes [8,26–28]. Beyond this timeframe, fat mass either returned to- or exceeded baseline measures prior to contest preparation between 9 weeks and 6 months post-contest [16,32–36]. Rapid weight gain (up to 9 kg) was commonly seen immediately post-contest within the first 4 weeks [26,27,33]. Lean body mass was maintained during this time [10,27,32], or in the case that lean mass was lost during contest preparation, lean mass was recovered within 2–5 months post-contest [34,36].

### 3.3 Dietary intake

Diet was observed in all case studies through weighed food records or meal diaries (83%, *n* = 10), or through the combination of a food diary and 24 hr diet recall (17%, *n* = 2). An immediate increase in energy intake was observed post-contest with ad libitum eating in 50% (*n* = 6) of case studies [7,26,27,32,35,36]. Longstrom et al. observed one female athlete who increased calorie intake post-contest by 97% coinciding with a 22% increase in fat mass and restoration of physiological function within 10 weeks [33]. A structured gradual increase in energy intake was a strategy observed in 42% (*n* = 5) of case studies where athletes followed various protocols to restore pre-contest baseline body fat percentages or to restore energy availability [16,28,33,34,37]. These protocols involved increasing energy availability in a stepwise manner with the target of increasing body mass by 1% per week [16], or by increasing calorie intake gradually, as seen by Pardue et al. where carbohydrate and fat intake were increased by 10–30 g and/or 4–10 g, respectively, each week of the recovery phase up to five months post-contest [34]. These structured gradual approaches to increasing energy availability were observed between 9 weeks and 5 months post-contest with high variability between individuals [16,33,34,37]. Protein intake commonly remained above 2 g.kg^−1^ throughout the duration of the post-contest recovery phase [36,37].

### 3.4 Resting metabolic rate (RMR)

RMR was observed in 59% of the total participants (*n* = 41) using indirect calorimetry. Estimated energy availability using the following equation (energy intake (kcals)-EEE (kcals))/fat-free mass (FFM; kg), was an alternative method used by Halliday et al. to assess changes to metabolic rate in a female athlete [37]. For studies following athletes less than 2 months post-contest, 24 athletes saw no significant change in RMR, and two female athletes increased RMR or returned to pre-contest baseline RMR levels within 4 weeks post-contest [16,33]. Additionally, Trexler et al. showed a positive association between RMR restoration and body fat percentage between the pre-contest preparation phase and 4 to 6 weeks post-contest [26]. Studies observing athletes for 2 to 6 months found 13 out of 15 participants experienced an increase in RMR or energy availability, toward or achieving pre-contest preparation baseline measures [8,33–37]. Participants who did not see an increase during this time either experienced RMR suppression or no significant change (*n* = 2) suspected due to exercise induced amenorrhea [8,33]. Within this time, two athletes exceeded pre-contest baseline RMR measures by 5–8% [36,37].

### 3.5 Endocrine measures

Endocrine measures were observed in 75% (*n* = 9) of case studies via blood serum (*n* = 7), saliva samples (*n* = 1), or a mixture of the two (*n* = 1). The most common endocrine measures performed on participants included testosterone (*n* = 58), thyroid hormones including T4, T3, and/or TSH (*n* = 37), leptin (n = 61), cortisol (*n* = 53), ghrelin (n = 17), sex hormone-binding globulin (SHBG) (*n* = 10), insulin (*n* = 25), and estradiol (*n* = 28). Testosterone increased toward baseline measures pre-contest preparation or reached baseline within 4 to 6 weeks post-contest across the most male participants (*n* = 17) [26,27,36]. Two male physique athletes were observed to require up to 6 months post-contest to recover testosterone levels [34,35]. Testosterone remained below pre-contest baseline measures in most female athletes (*n* = 35) observed up to 4 months post-contest [26,32]. Ghrelin and thyroid hormones were observed to increase toward pre-contest baseline measures, although they did not fully reach baseline until up to 6 months post-contest [32–35]. Leptin was observed to increase toward or reach pre-contest baseline measures within 4 months post-contest, however it was also shown to remain below baseline measures up to the final data point at 6 months [27,32,33]. Cortisol decreased within 4 to 6 weeks post-contest, which was previously elevated within the weeks leading up to contest day [26,27]. SHBG and insulin increased or returned to baseline within 4–6 weeks post-contest [26,27,36]. Estradiol was monitored in only one female athlete post-contest, with measures returning to pre-contest baseline levels within 4 months post-contest [32].

### 3.6 Menstrual function

Menstrual function was tracked via questionnaires in four case studies. Across 33 female athletes, most experienced menstrual irregularities across individual study timeframes (*n* = 31) [16,32,33,37]. Hulmi et al. reported 7 out of 27 athletes experiencing amenorrhea up to 4 months post-contest [32]. Halliday et al. observed one athlete experiencing the return of menses 71 weeks post-contest [37]. Normalized menstrual function within 10 weeks post-contest was observed in one female athlete coinciding with the greatest increase in energy intake, body mass, and fat mass [33].

### 3.7 Psychological aspects of recovery

Psychological aspects were observed in five case studies using a range of questionnaires, such as the Eating Attitude 26-item questionnaire (EAT-26), the Profile of Mood States questionnaire (POMS), and other body image and anxiety scales, such as the Body Appreciation Scale (BAS-2), Social Physique Anxiety Scale (SPAS), and the Perceived Stress Scale (PSS) [8,28,35]. Anxiety, weight phobia, compulsive self-monitoring, body image concern, and total mood disturbance was generally greatest immediately post-contest and within the first 4 to 8 weeks post-contest [8,28,33,35]. Psychometric scores trended toward baseline within 6 months post-contest [33,35].

## 4. Discussion

Among the research articles included in this review, there were six common physiological parameters of concern arising from contest preparation. These were body composition, dietary intake, RMR, endocrine measures, menstrual cycle irregularities, and psychological aspects. During the post-contest recovery phase, these six parameters tended to return to baseline measures within six-month post-contest; however, RMR, endocrine measures, menstrual function, and psychological measures may take longer to achieve full recovery for some athletes.

### 4.1 Body composition

In the post-contest recovery phase, it is common for physique athletes to experience body composition changes associated with rapid weight gain. These changes in body composition are likely associated with fat gain due to diminished RMR and hedonic responses to food, such as extreme hunger, hyperphagia, and binge eating that are often observed during the recovery period [7]. Such a phenomenon is contrary to the goals of minimizing body fat overshooting beyond pre-contest baseline measures and assisting preparation for future physique contests [2,34]. However, favoring fat regain may be necessary for physique athletes as persistent low body fat in both sexes has been known to interfere with the recovery of physiological outcomes, leading to further disruptions in RMR and sex hormones [7,17]. Three case studies observed that athletes typically return to baseline body fat levels within 2–6 months post-contest [16,35,36]. Roberts et al. suggest a faster return to baseline body fat levels within 1–2 months post-contest is preferential for full physiological recovery [7]. This indicates that although body fat regain could be contrary to the athletes’ objectivesfor example, bypassing pre-contest baseline levels (fat overshooting), it is likely a fundamental part of the recovery period for an athlete’s full physiological recovery post-contest. Hence, a strategic return to baseline body fat levels is imperative to optimize health and future training performance.

A gradual increase in total body mass of 1% per week post-contest is another strategy used to return to baseline body fat levels and minimize fat overshooting while facilitating physiological recovery [16,26]. Following this strategy, Tinsley et al. showed a return to baseline body composition and RMR by 9 weeks post-contest along with a reduction in uncontrolled eating behaviors [16]. However, in some athletes, these uncontrolled eating behaviors driven by hedonic responses to food could in turn be an impediment to this gradual approach. Hedonic responses to food may persist beyond baseline body fat restoration, hence promoting excess fat accumulation, or fat overshooting, and weight gain until lean mass is recovered [38,39]. During contest preparation, energy availability is generally not sufficient to support muscle hypertrophy [21], hence maintaining muscle mass during contest preparation becomes a key factor to minimize fat overshooting post-contest. To achieve muscle maintenance, an athlete’s training is the main modifiable factor to support muscle mass, followed closely by adequate protein intake [9]. As a result, the importance of a structured resistance training program extends beyond the preparation phase and into the recovery phase to support lean mass restoration. Therefore, post-contest restoration of lean mass requires the continuation of a structured resistance training program, increased energy intake, and adequate protein intake to support muscle growth and minimize fat overshooting. Such recommendations may minimize post-contest hedonic responses to food and body fat overshooting, facilitating full physiological recovery.

### 4.2 Dietary intake

All case studies observed dietary intake of physique athletes transitioning from a negative to positive energy balance post-contest. Athletes chose to either implement a structured gradual increase of energy intake, termed “reverse dieting,” or alternatively an ad libitum energy intake strategy immediately post-contest, often linked to compulsive eating and hyperphagia [7,33–35]. Dietary decisions were made by athletes and/or coaches, and for those not following an ad libitum approach, gradual increases in dietary intake were determined by coinciding with increases in body weight [16]. Although both strategies could be used in the recovery phase as preferred by the athlete, this review demonstrates that there is a gap regarding evidence-based recommendations to increase energy intake post-contest. Therefore, dietary recommendations for individuals post-contest should be considered with caution and explored at the discretion of the athlete and coaches.

An acute increase in energy intake immediately post-contest is a common ad libitum eating practice described by physique athletes as it lines up with an athlete’s refeeding impulses experienced post-contest [12,26]. Such sharp increases in energy intake may have favorable effects contributing to RMR restoration, as suggested by Trexler et al., where RMR increased 5.3% above predicted values within 6 weeks post-contest alongside a 90% increase in energy intake, which was also correlated to an increased body fat and high-protein intake [26]. Similarly, less structured ad libitum dietary strategies to increase energy intake post-contest have been shown to restore hormones associated with hunger (leptin, ghrelin, insulin) in as little as 3 weeks post-contest [7]. However, it is also noted that these strategies may contribute to body fat overshooting and undesirable body composition outcomes for the athletes, mostly when prolonged above maintenance energy intake [7]. Therefore, ad libitum increases in energy intake after a period of LEA, such as the post-contest recovery phase, may be more beneficial as an acute strategy to satiate refeeding impulses experienced by the athlete [17]. This could then be followed by a more structured approach to restoring energy availability by either returning to maintaining energy intake or gradually increasing energy intake toward maintenance. Prolonging ad libitum intake beyond 1 to 2 weeks post-contest should be considered on an individual basis, bearing in mind the athlete’s body composition goals, psychological state, and requirements for physiological recovery.

The second strategy of structuring a gradual approach to increase energy intake post-contest (reverse dieting), may offer an energy restoration strategy to minimize fat overshooting while facilitating physiological recovery [33,34]. This strategy involves maintaining a high protein intake (above 2 g.kg^−1^), alongside a gradual increase in carbohydrates and fat (e.g. 10–30 g carbohydrate and/or 4–10 g fat added per week) to return body fat and lean mass to pre-contest baseline levels [16,34,37]. This dietary approach may also coincide with a stepwise reduction in aerobic exercise to facilitate restoration of energy availability [34]. However, despite anecdotes in the gray literature, reverse dieting remains a theoretical concept and this approach might slow physiological recovery as it prolongs fat restoration [33]. While following a reverse dieting strategy, participants with minimal weight gain showed the least improvements to RMR and endocrine measures as opposed to one female who immediately increased intake by 97% and achieved a 22% increase in body mass alongside full RMR recovery and menstrual function restoration [33]. Although this structured gradual approach to increased energy intake has potential benefits for athlete body composition, the recovery phase will require a longer time frame for full physiological recovery in comparison to an acute increase in energy availability and body fat restoration. Furthermore, based on the available literature and anecdotal evidence, in practice there are barriers regarding adherence to controlled reverse dieting post-contest due to diet fatigue experienced after 16-plus weeks of contest preparation and restrictive eating [2]. Therefore, another proposed method of restoring energy availability post-contest could involve an immediate return to maintaining energy intake. Athletes and coaches should consider adequate macronutrient intake, prioritizing protein, and continuing a structured resistance training program while implementing a gradual reduction of aerobic exercise to support full physiological restoration throughout the recovery phase.

### 4.3 Resting metabolic rate (RMR)

A small number of case studies directly monitored RMR in physique athletes using indirect calorimetry. Although indirect calorimetry is a valid method for measuring RMR, inaccuracies often occur in athletic populations, including physique athletes, where training regimes may inflate RMR for up to 48 hours after training [40]. This was reflected in a study by Mitchell et al., where no change in RMR was observed in physique athletes pre- and post-contest, despite fluctuations in body composition and sex hormones, which was suggested to be an inaccurate measure associated with high-frequency training regimes [27]. Restoration of RMR was found to be associated with the recovery of other aspects of the athlete’s physiology (i.e. body composition and endocrine function), therefore monitoring RMR restoration alongside other physiological measures supports interpretation of RMR fluctuations [26,33]. It is recommended that future research measures RMR pre- and post-contest, while considering the limitations of indirect calorimetry and aim to minimize the confounding effect of an athlete’s training program on RMR accuracy.

Reductions in RMR from baseline pre-contest levels to contest day are commonly seen in physique athletes [26]. Although highly variable across individuals, physique athletes can experience reductions in RMR as low as 20% below baseline levels at the end of the preparation phase [36]. Furthermore, how fast weight is lost during the preparation phase may have a direct impact on reductions in RMR. This has been previously shown in a systematic review looking at overweight and obese individuals undergoing gradual versus rapid weight loss where greater reductions in RMR were seen during rapid weight loss [41]. Physique athletes may experience similar downregulations of RMR, depending on their contest preparation strategies. Further, heterogeneity of contest preparation strategies used by athletes could be a major contributor of observed RMR variability. Regardless of the reduction of RMR observed, the time window required for RMR recovery post-contest is largely unknown. Full reversal of suppressed RMR took place in as little as 4 to 6 weeks post-contest in some athletes, while others required beyond 6 months [8,16,33,36]. Body fat reduction achieved during contest preparation, alongside energy restriction and potential muscle catabolism, is known to directly downregulate RMR [42]. Recovery of RMR is likely intertwined with some degree of fat mass and fat-free mass restoration [7]. Likewise, increased energy availability and high protein intake may play a role in the recovery of RMR [26]. Anecdotally, there is a common belief that greater muscle mass can yield a higher RMR [43]. However, in physique athletes, muscle mass alone has shown little to no effect on RMR preservation throughout contest preparation [7,36]. Instead, RMR restoration post-contest is likely associated with factors other than muscle mass, such as refeeding strategies, energy availability, and total body mass and fat restoration [7,36]. This suggests that RMR restoration strategies should focus on increasing energy availability and total body mass.

### 4.4 Endocrine measures

A high degree of attention in the literature is given to hormonal disruptions in both male and female physique athletes, mainly immediately before and after the contest. In particular, the synthesis and function of sex hormones (testosterone and estrogen), thyroid hormones (T3 and T4), and hunger and energy balance hormones (leptin, ghrelin, insulin, and cortisol), fluctuate in response to physiological changes during contest preparation. Nine studies in this review evaluated testosterone and/or leptin, and less than five studies observed thyroid measures, cortisol, insulin, and/or ghrelin. Despite the apparent increasing popularity of hormone investigations within physique athletes’ post-contest studies, there is currently insufficient evidence outlining the time in which full restoration of hormonal function occurs. Some studies suggest as little as 4 weeks to recover hormonal function and others demonstrate a continuous increase in hormone levels toward baseline beyond 6 months post-contest [34,35]. Such pronounced discrepancies are suspected to be due to individual variations regarding energy availability and fat mass restoration, as well as the inter-study variability in measured hormonal parameters, perhaps as a result of resource availability and time limitations [26]. The heterogeneity of the data included within the reviewed articles hinders the ability to interpret the rate of recovery and the following comments should be considered with care.

There is a variety of hormones that are especially relevant to the physiological recovery of physique athletes, including hunger hormones such as leptin, ghrelin, and insulin, and endocrine hormones such as estrogen, testosterone, and thyroid hormones. Similar to RMR, transitioning from a negative to positive energy balance post-contest is likely to have immediate favorable effects on leptin, ghrelin, and insulin [44]. The full recovery of hormone measures is likely influenced by fat mass and total weight restoration, as well as energy availability, and the continuation of extreme exercise regimes [7,8,20,32–34]. As a result, prolonged downregulation of hormones often occurs in athletic populations, where LEA and chronically high physical activity levels are commonly experienced [20]. This is seen in physique athletes post-contest who remain at low body fat levels or maintain extreme exercise regimes, likely perpetuating the suppression of leptin and leading to a cascade of effects on thyroid hormones and the reproductive and growth hormone axis [20,26,34]. Considering the available evidence, to minimize the unfavorable effects of hormonal downregulation, it is suggested that physique athletes promptly transition to a positive energy balance post-contest via an increase in energy intake and a reduction in exercise demands.

### 4.5 Menstrual function

Articles included in this review were relatively evenly focused on male and female subjects, which is useful as females are typically under-represented in sport science research [45]. However, across the studies reviewed, only one study observed female sex hormones, of which estrogen returned to baseline measures by 4 months post-contest [32]. This aligned with menstrual cycle irregularities and amenorrhea experienced by participants during this time frame [32], which tend to be more prominent in physique contest categories that require lower levels of fat mass, such as figure and bodybuilding categories [7]. These hormonal disruptions contribute to rapid weight regain, often referred to as weight cycling or fat overshooting, by driving compulsive eating and hyperphagia, mood disturbances, reduced energy expenditure or reduced exercise performance, and the onset of eating disorders and other negative physiological impacts [42]. Given the wide spectrum of physiological disruptions, and their adverse outcomes common to this sport, such as menstrual cycle irregularities, further investigation into evidence-based approaches to minimize these effects is imperative.

Considering the regular occurrence of menstrual irregularities and amenorrhea in female physique athletes, the study of female sex hormones in this population represents a substantial gap in knowledge that warrants further investigation [16,32,33]. Halliday et al. demonstrated that menstrual function restoration can take up to 71 weeks post-contest, independent of energy availability and body composition returning to baseline levels in half of that time [37]. The variability of menstrual cycle recovery is individual to the athlete and interlinked with energy availability, body mass, eating behaviors, and endocrine factors [46]. Longstrom et al. observed that menstrual function restoration may be possible within 10 weeks post-contest through a rapid increase in energy intake and fat mass restoration [33]. However, these findings could also be related to increases in visceral fat mass, suggesting that diminished fat mass around internal organs play a role in downregulating female sex hormones involved in ovulation and menstrual bleeding [32]. Increased energy availability immediately post-contest and visceral fat restoration may be advantageous for menstrual cycle recovery post-contest, although further research is required.

### 4.6 Psychological aspects of recovery

Studying psychological measures within the context of dietary manipulation and pronounced changes in body composition as seen in physique athletes, is imperative due to the entwined nature of eating behaviors, body image, and their impact on physiological recovery post-contest. Across the reviewed articles, the methodology used to assess psychological aspects varied, including eating behavior questionnaires, body image and anxiety measures, and other validated surveys. The current evidence reports an increase in uncontrollable eating behaviors, hyperphagia, and mood disturbances in the early phases of recovery [7,16,35,36]. These responses tend to shift toward baseline levels 2–6 months post-contest, presumably coinciding with the full physiological recovery of the athlete [16,35]. The inconsistency in approaches to assessing psychological aspects of post-contest recovery in these athletes limits the capacity to draw conclusions, and consequently, the psychological aspects of post-contest recovery in physique athletes remain largely unexplored. Hence, researchers are encouraged to include validated measures of psychological wellbeing in future studies. Individual nutritional recommendations for post-contest recovery should consider strategies that are in the best interest of psychological health.

### 4.7 Applications to other areas of sport

Physique athletes provide insight into the negative impacts of extreme dieting and how dietitians, sports scientists, and other health-care professionals can facilitate full physiological recovery from such physiological states. Relative Energy Deficiency in Sport (RED-S) and LEA are the most commonly studied across athletic populations, such as endurance athletes and weight-making sports [47]. However, these issues can also occur in non-athletic populations where societal pressures and body image perceptions drive dietary restrictions and states of LEA [48]. Gradual weight loss (0.5 to 1.0 kg per week) approaches have been deemed appropriate across both athletic and non-athletic populations to minimize the negative effects on RMR and optimize sustainable body fat loss, as opposed to rapid weight loss [9,41]. However, regardless of the weight loss strategy, adverse physiological outcomes still exist when individuals experience a sustained energy deficit and significant body fat loss [7,10]. Post-diet recovery strategies to mitigate the risk of ongoing negative physiological adaptations related to LEA and RED-S are essential for long-term maintenance of results while also supporting optimal physiological function. Investigations into the physique of athletes present an opportunistic case for unraveling recovery strategies post-dieting.

### 4.8 Limitations

The understanding of post-contest recovery for physique athletes is predominantly informed by a small number of heterogeneous case studies and case series. Most studies followed only 1 athlete (50%) or between 2 and 15 athletes (42%), and recovery phases observed were overall short; often less than 10 weeks long [8,16,26–28,33]. One study following a female physique athlete reported complete physiological recovery occurred at 71 weeks [37], approximately seven times more than the average recovery length assessed by current studies. A longer period of observation might be needed along with a larger sample size to provide more robust recommendations. However, lengthy periods of observation could impact the accuracy of self-reported dietary intake and compliance. Also, physique athletes’ baseline physiology prior to contest preparation varied, that is, starting body fat percentage, occurrence of low leptin levels, and menstrual irregularities [36,37]. These variabilities may mislead the interpretation of post-contest recovery, therefore future research should aim to collect participants with similar baseline physiology.

## 5. Conclusion

### 5.1 Recommendations for post-contest recovery

Recommendations for physique athletes preparing for contest day are relatively well established. Less is known about post-contest recovery, as the current literature is limited to observational case studies with small sample sizes and varied methods for observing physiological and psychological outcomes. The limited body of evidence suggests a gradual return to baseline measures occurs for body composition, RMR, and endocrine measures during post-contest recovery, so long as the athlete increases energy availability and body fat mass. These measures tend to occur over a period of six months with high levels of variability within individuals and potentially requiring longer recovery times.

Dietary strategies to facilitate physiological restoration in these athletes are unclear. Potential strategies to further explore are: (1) a structured gradual increase in dietary intake aimed at reaching maintenance energy levels; (2) an acute ad libitum increase in dietary intake immediately post-contest to facilitate continued dietary adherence, followed by a structured gradual increase in intake toward maintenance energy levels; or (3) an immediate return to maintenance dietary intake. Based on the available evidence, it is recommended that any of these strategies be implemented alongside a high protein intake and strategically managing training type and load. It remains to be seen which of these dietary strategies are preferential to restore baseline physiology.

### 5.2 Recommendations for future research

There remains a large gap in our understanding of post-contest recovery for physique athletes. Future research should consider intervention studies to compare various post-contest dietary strategies using randomized trials with a controlled manipulation of energy restoration post-contest. Study designs require a balance between observation lengths to fully collect recovery data while minimizing the burden associated with lengthy data collection periods.

## List of abbreviations

RMR: resting metabolic rate
LEA: low energy availability
CHO: carbohydrate
LBM: lean body mass
BM: body mass
FFM: fat free mass
BF: body fat
HR: heart rate
BP: blood pressure
T: testosterone
E2: estradiol
C: cortisol
SHBG: sex hormone binding globulin
T3: triiodothyronine
T4: thyroxine
Mg: magnesium
AST: aspartate transaminase
ALT: alanine aminotransferase
BUN: blood urea nitrogen
WBC: white blood cell count
AGR: albumin globulin ratio
IGF-1: insulin-like growth factor-1
LFT: liver function tests
CK: creatine kinase
FM: fat mass
EA: energy availability
EP: exercise performance
AAS: androgenic anabolic steroids.
